# The Association Between Maternal Dietary Intake and the Risk of Heavy Metals in Human Breast Milk in Korea

**DOI:** 10.3390/toxics13050381

**Published:** 2025-05-08

**Authors:** Nalae Moon, Su Ji Heo, Seungyoung Park, Hosub Im, Ju Hee Kim

**Affiliations:** 1 Department of Nursing, College of Nursing Science, Kyung Hee University, Seoul 02447, Republic of Korea; mnl0302@khu.ac.kr (N.M.); tnwl0428@khu.ac.kr (S.J.H.); 2 Food Safety Risk Assessment Division, Food Safety Evaluation Department, National Institute of Food and Drug Safety Evaluation, Osong Health Technology Administration Complex, Cheongju-si 28159, Republic of Korea; sypark0307@korea.kr; 3 Institute for Life & Environmental Technology, Smartive Corporation, Hanam-si 12902, Republic of Korea; hosublim@gmail.com

**Keywords:** heavy metals, breast milk, breastfeeding, diet, risk assessment

## Abstract

Heavy metals (HMs) persist in the environment and enter the human body via various pathways. Once stored in adipose tissue, they can be transferred to breast milk, posing risks to infants. Moreover, maternal diet plays a key role in influencing HM levels in breast milk. The aim of this study was to measure the concentration of HMs, including lead (Pb), cadmium (Cd), mercury (Hg), and arsenic (As), in the breast milk of Korean mothers, assess their potential health risks, and identify maternal dietary factors influencing HM concentration in breast milk. Survey data on maternal–infant pairs and breast milk samples from 209 healthy lactating mothers were collected between January and March 2023. Trained nurses manually expressed the breast milk to prevent external contamination, and maternal dietary intake was systematically assessed using the 24 h recall method. The concentrations of Pb, Cd, Hg, and As were measured, and a risk assessment was conducted using the estimated daily intake hazard quotient. A multiple linear regression model was employed to evaluate the association between the HMs in breast milk and maternal dietary factors. Cd was detected in 99% of the breast milk samples, and high detection rates were observed for Hg (97%), As (89%), and Pb (79%). Arsenic was the largest contributor to the potential health risks of HM-contaminated breast milk. Positive associations were observed between Pb concentration and legume and seaweed consumption, Cd concentration and vegetable and seaweed consumption, Hg concentration and sugar intake, and As concentration and meat intake. This study highlights that maternal dietary intake is closely linked to HM concentrations in breast milk, and elevated As levels potentially pose health risks to infants. These findings underscore the importance of adopting a healthy diet to reduce HM exposure in breast milk and to promote safer breastfeeding practices.

## 1. Introduction

Although occurring in nature in trace quantities, heavy metals (HMs), including Pb, Cd, Hg, and As, are well known for their potential toxicity to humans, especially to vulnerable populations [1]. While As is technically a metalloid rather than a metal, it is treated as part of the heavy metal group in this study due to its comparable toxicological characteristics and relevance in infant exposure through breast milk [1]. For the purposes of this study, we adopt a practical classification that includes As among heavy metals to better reflect the real-world risks associated with dietary exposure in infants. Infants are particularly susceptible to HM exposure because their rapid growth, developing nervous system, and immature blood–brain barrier, plasma protein–binding capacity, and enzyme elimination systems in the liver and kidneys reduce their ability to metabolize and excrete HMs [1,2]. Previous studies have demonstrated that HM exposure can negatively affect neurological development, resulting in lower intelligence quotient (IQ) and, thus, learning difficulties in infants and young children [2,3,4,5,6]. Recognizing these potential risks of HM exposure to infants and young children, several countries are implementing policies aimed at reducing HM exposure. For example, the “Closer to Zero” initiative has been implemented in the United States of America (USA) to reduce HM levels in food products consumed by infants and young children [7]. Similarly, the European Union (EU) has implemented the “As Low As Reasonably Achievable” system to regulate HM exposure [8]. Furthermore, national and international agencies are continuously reassessing and strengthening regulations related to HMs. For instance, a recent study in the USA highlighted concerns regarding Cd exposure through cocoa products, resulting in stricter global regulations on cocoa [9]. Consequently, the Korean Ministry of Food and Drug Safety (MFDS) has reviewed new standards for the Cd content of cocoa products since 2024 [10].

HMs are lipophilic and bio-accumulative; due to their tendency to be stored in the adipose tissue in the breast, HMs can be transferred to breast milk through either passive diffusion or active transportation [11]. On the other hand, Pb can accumulate in maternal bones along with calcium (Ca) to supply sufficient Ca to the fetus; thus, accumulated Pb can be excreted along with Ca ions [12]. Therefore, breast milk is a valuable biological matrix for understanding how both mothers and infants are exposed to HMs [12]. Several previous studies have reported the presence of HMs in human breast milk, with some studies indicating that the hazard quotient (HQ) exceeded the risk threshold. According to a study by [13], the carcinogenic risk associated with infant exposure through breastfeeding surpassed the safety level suggested by the United States Environmental Protection Agency (EPA), with As being the most significant contributor. Furthermore, in an analysis of over 200 breast milk samples, Hg was detected in 100% of samples, while Pb was found in 77%, and more than 50% of infants under 30 days old had an HI exceeding 1 [14]. Some studies have attempted to identify sources of heavy metal exposure by examining maternal diet, lifestyle factors, and contamination levels in regionally available foods [15,16,17,18]. These studies suggested that higher consumption of grains, frequent exposure to cosmetics or paints, and the intake of tap water were associated with elevated heavy metal concentrations in breast milk [15,16,17,18]. Similarly, numerous studies have identified a consistent relationship between dietary intake—including specific foods such as fish and vegetables—and mercury levels in adult blood [19,20,21,22]. Maternal blood levels of heavy metals are generally known to be associated with concentrations in breast milk, as many metals can cross and accumulate in breast tissue and be excreted through lactation. This biological link reinforces the relevance of maternal biomarkers in assessing infant exposure via breastfeeding [16,17,18].

However, several limitations exist in the current body of research. First, the estimation of infant exposure levels through breastfeeding often relies on average intake and body weight, with most studies using a single representative value rather than accounting for monthly variations in infant age [13,17,18,23,24]. This approach fails to accurately reflect age-specific changes in body weight and milk consumption, potentially leading to overestimation or underestimation of risk assessment outcomes. Additionally, previous studies have either not investigated the sources contributing to heavy metal accumulation in breast milk or lacked methodological precision. Many studies did not assess exposure sources at all [13,14,17,23,24], while others measured only intake frequency rather than actual dietary intake records [16] or analyzed contamination levels in local food products rather than assessing individual exposure levels [18]. Furthermore, some studies had extremely small sample sizes, with fewer than 10 breast milk samples analyzed [15], while others focused on only one or two selected HMs rather than a comprehensive evaluation [14,15].

Given the presence of HMs in breast milk and their potential health risks to infants, the question still arises whether breastfeeding remains the optimal choice compared to formula feeding. Since dietary intake is a primary route of human exposure to HMs [25], maintaining a well-balanced maternal diet may help mitigate the transfer of these contaminants to infants through breast milk. Previous research has highlighted that maternal dietary habits influence heavy metal concentrations in breast milk. For instance, a Japanese cohort study found that a balanced diet was associated with lower maternal Pb and Cd levels but higher Hg concentrations [26]. Similarly, a Spanish study reported that mothers consuming large quantities of potatoes during lactation exhibited increased Pb levels in their breast milk, underscoring the role of diet in heavy metal accumulation and excretion through lactation [27].

While prior studies have acknowledged the dietary influence on breast milk heavy metal content, they often lacked precision in exposure assessment, did not comprehensively account for monthly variations in infant intake and body weight, or were limited by small sample sizes. To address these gaps, this study not only measures heavy metal concentrations in breast milk but also assesses the associated health risks by incorporating a refined exposure assessment model that accounts for infant age-specific intake patterns. Additionally, by analyzing comprehensive dietary records of mothers, this study aims to identify the specific maternal dietary factors that contribute to heavy metal concentrations in breast milk. Through a robust exposure assessment framework, this study provides a more accurate characterization of infant risk while also offering evidence-based insights into dietary modifications that may help reduce heavy metal transfer through breastfeeding.

## 2. Materials and Methods

### 2.1. Study Population and Data Collection

This study is part of a prospective cohort study aiming to determine the impact of early life exposure to environmental hazards, including HMs, on infant and child health in Korea from 2021 to 2024; baseline surveys were conducted from the pregnancy period to 3 years after birth (infant and toddler period), with multiple follow-up periods [28]. Eligible pregnant women were enrolled into the cohort beginning in March 2021. Urine and breast milk samples were repeatedly collected during pregnancy and postpartum to assess exposure to endocrine-disrupting chemicals. Participants were proportionally allocated based on 2019 national birth statistics to ensure geographic representativeness. Recruitment notices were disseminated through regional obstetric clinics, and women who voluntarily contacted the research team were enrolled following confirmation of eligibility. All participants were informed of the purpose of this study, and only those who provided written consent were included in this study. From 5 January to 15 March 2023, data and breast milk were collected from 209 healthy mother–infant pairs included in the cohort study. The inclusion criteria were as follows: (1) healthy mothers who were breastfeeding their infants during the study period and (2) healthy mothers who had infants less than 12 months old. Mothers who had mastitis or other breast problems were excluded from data collection. This study was approved by the Institutional Review Board of Kyung Hee University [KHSIRB-24-218(NA) and KHSIRB-21-598(NA)].

The questionnaire employed in this study was developed based on previous research on the association between persistent and non-persistent endocrine-disrupting chemicals and dietary intake; it was validated by experts in environmental epidemiology, toxicology, and food nutrition [29,30]. The items included in the questionnaire were the sociodemographic characteristics of mothers (age, height, weight, education, employment, household income, place of residence, and parity) and infants (age, sex, weight, height, birth weeks, and birth weight), and breast milk consumption and maternal dietary intake. An electronic scale (Hubidic Digital Infant Scale Height Scale HUS-315B, Anyang-si, Korea) was used by a registered nurse to directly measure the height and weight of the infants, who wore only a minimum amount of thin clothing without diapers. To collect breast milk samples safely while minimizing contamination from external sources, qualified registered nurses employed the hand-pressing method to collect 20 mL of breast milk into pre-cleaned glass bottles and immediately stored these at −70 °C until laboratory analysis was performed.

### 2.2. Risk Assessment of HMs in Breast Milk

The process of assessing the risk of chemicals is divided into the following stages: (1) hazard identification, (2) hazard characterization, (3) exposure assessment, and (4) risk characterization. In the exposure assessment process, the estimated daily intake (EDI) posed by HMs (Pb, Cd, Hg, and As) in breast milk to infants was calculated as follows:

EDI = (C × DI)/BW (μg/kg bw day)(1)

where C is the concentration of the analytes in breast milk samples (μg/L), DI is the amount of daily breast milk intake (frequency × amount of one-time breastfeeding) of each infant (L/day), and BW is the body weight (kg) of each infant.

In the risk characterization process for HMs, we evaluated the potential risk to human health by obtaining HQ values. HQ is the ratio of EDI to the threshold values of the target HMs and is calculated by dividing EDI by the threshold value. An HQ value > 1 implies a potential health risk of HMs due to dietary intake. We referred to the threshold value suggested by the Joint FAO/WHO Expert Committee on Food Additives (JECFA) and the Korean MFDS for threshold values [10,31,32,33,34] (Table 1).

### 2.3. Dietary Intake Measurements

The breast milk intake of the infants was measured using the test-weighting method, wherein an infant’s weight was measured before and after breastfeeding; the difference between the initial and final weight is considered the intake [35]. An auxiliary method was also employed. For the mothers who used a breast pump, the amount of breast milk was calculated by expressing it with a breast pump in advance, while for the mothers who did not use a breast pump, the amount of milk was estimated by hand-expressing the milk and putting it in a bottle.

Maternal dietary intake was measured using a 24 h recall method for 2 non-consecutive days. The effectiveness of this method depends on the participants’ ability to accurately recall their dietary intake and the interviewer’s skills in eliciting complete and detailed information [36]. Therefore, the mothers were trained to write down and take pictures, if necessary, of the types and amount of all food and drinks they consumed for 2 non-consecutive days. Afterwards, trained investigators rechecked food quantities using 2D/3D tools, such as marked bowls and spoons. Raw data of maternal dietary intake were extracted and analyzed by nutritional experts using a nutritional analysis program (CAN-Pro 5.0 Web version; The Korean Nutrition Society, Seoul, Korea). Data on the foods consumed by the participants were divided into 16 food groups: grains, potatoes and starch, sugars, legumes, seeds and nuts, vegetables, mushrooms, fruits, meats, eggs, fish and seafood, seaweeds, dairy products, fat and oils, beverages, and condiments. The concentrations of HMs in the food consumed by the mothers were determined using the latest contamination data provided by the Korean MFDS in 2024 [10].

### 2.4. Instrument Analysis

The direct alkali dilution method, based on the approach outlined by a previous study [37], was employed to quantify Pb, Cd, and total As (tAs) in the breast milk samples because it is known for its efficiency and accuracy in detecting trace metals in complex biological matrices such as breast milk. Approximately 0.1–0.2 g of each breast milk sample was accurately weighed and diluted with an alkaline solution containing ammonium hydroxide or sodium hydroxide, which help in stabilizing metal ions in the solution; the dilution factor ranged from 25 to 35 times the original volume of the breast milk samples. This step is crucial for minimizing matrix effects and bringing the HM concentrations within the optimal detection range of the analytical instrument. Afterwards, the samples were sonicated for 5 min to break down any residual fat globules and ensure that HMs were uniformly distributed within the samples. Subsequently, the samples were centrifuged at 2000 rpm for 5 min to separate any particulate matter, ensuring that the supernatant containing dissolved HMs was easy to analyze. The supernatant was subjected to inductively coupled plasma mass spectrometry (ICP-MS) to analyze its HM content. Specifically, a Nexion 2000 B ICP-MS (PerkinElmer, Waltham, MA, USA) in dynamic reaction cell (DRC) mode, which is particularly advantageous for reducing potential interference from polyatomic ions that may arise during the analysis of complex biological matrices, was utilized. The detailed operating conditions for ICP-MS, such as plasma power, nebulizer gas flow rate, and collision/reaction cell gases, are provided in Supplementary Table S1. These conditions were optimized to enhance the sensitivity, accuracy, and reliability of HM detection and quantification even in trace quantities.

A different approach was adopted for the analysis of Hg because of its unique properties, particularly its volatility and tendency to exist in various oxidation states. A direct mercury analyzer (DMA; Milestones Srl, Sorisole, Italy) was used for rapid and direct quantification of Hg without the need for complex sample preparation or chemical digestion. Approximately 100 μL of each breast milk sample was placed directly in a sample boat without any prior digestion. The samples were then subjected to thermal decomposition, amalgamation, and atomic absorption spectroscopy using the DMA system.

### 2.5. Quality Control and Quality Assurance

To ensure the accuracy and reliability of the HM analysis, rigorous quality control (QC) and quality assurance (QA) measures were implemented. The limits of detection (LODs) for Pb, Cd, Hg, and As were determined using the standard deviation (σ) of multiple blank sample measurements and the slope of the calibration curve, applying the formula LOD = 3σ/slope. Here, σ represents the standard deviation of blank analyses, while the slope corresponds to the response of the calibration curve. The calculated LOD values were 0.017 µg/L for Pb, 0.030 µg/L for Cd, 0.047 µg/L for Hg, and 0.054 µg/L for As. The accuracy of the analytical methods was assessed using the certified human milk reference material National Institute of Standards & Technology (NIST) SRM 1953 (Organic Contaminants in Non-Fortified Human Milk) from the (NIST, Gaithersburg, MD, USA). The reference material was analyzed in quadruplicate, and the measured concentrations were compared with the certified values to evaluate method accuracy. The deviation between the measured and certified values for validated elements remained within X%, confirming the reliability of the method. However, as NIST SRM 1953 does not provide certified values for Pb, Cd, and tAs, accuracy for these elements was evaluated through spiked recovery tests, where known concentrations (ranging from X to Y µg/L) of standard solutions were added to breast milk samples prior to analysis. The recovery rates for Pb, Cd, Hg, and As ranged from 92.5% to 105.3%, falling within the acceptable validation range. Method precision was assessed based on the relative standard deviation (RSD%) of replicate analyses, with RSD values for all HMs remaining below 5%, demonstrating strong repeatability and reliability. Method validation further included an evaluation of linearity (R^2^ > 0.99 for all analytes), sensitivity, and potential matrix effects using spiked and unspiked samples, ensuring the robustness of the analytical approach. Any deviations from expected recovery rates were carefully examined, and if necessary, analytical protocols were refined to improve accuracy.

### 2.6. Statistical Analysis

Continuous data are reported in means and standard deviations (SD), whereas categorical data are reported in percentages. The values of the target analytes below the LOD were replaced by LOD/√2 [38]. The normal distribution of all chemical concentrations was determined using the Kolmogorov–Smirnov test. All HM concentrations were natural log-transformed to normalize the right-skewed distribution. Target HMs that exhibited a detection frequency >70% in the breast milk samples were included in the final statistical analysis. Pearson’s correlation coefficient was used to evaluate the correlation between the HM concentrations in the breast milk samples and dietary factors. Multiple linear regression was applied to determine the association between HM concentrations in breast milk and dietary intake after adjusting for covariates including maternal age, maternal body mass index (BMI), education, monthly household income, residence area, and parity. Statistical analyses were conducted using SPSS 28.0 (Chicago, IL, USA) and R 4.1.0 (R Development Core Team, Vienna, Austria), with a significance level of *p* < 0.05.

## 3. Results

### 3.1. Characteristics of Mother–Infant Pairs

The socioeconomic characteristics of the mothers and the physical traits of the infants are shown in Table 2. The average age of the mothers was 34.8 years old (range: 22–43). The mean maternal BMI was 23.23 kg/m^2^, and over 94% graduated from college (*n* = 198). Over 50% of the mothers earned more than USD 5000 per month (57.42%), had a job (50.24%), lived in a metropolitan area (Seoul or Gyeonggi) (53.11%), and had a child for the first time (primipara) (60.29%). The average age of the infants was 2.47 months (range: 0.1–5.67) and 51.7% were girls. The average weight, height, gestational age, and birth weight of the infants were 5.71 kg (range: 2.99–9.40), 58.81 cm (range: 49.00–84.00), 38.71 weeks (range: 34.4–41.2), and 3.19 kg (range: 2.18–4.38), respectively. The average daily breast milk intake of the absolutely breastfed infants was 883.54 mL (range: 420–1260).

### 3.2. Concentration and Risk Assessment Levels of HMs in Breast Milk

**Figure 1:**
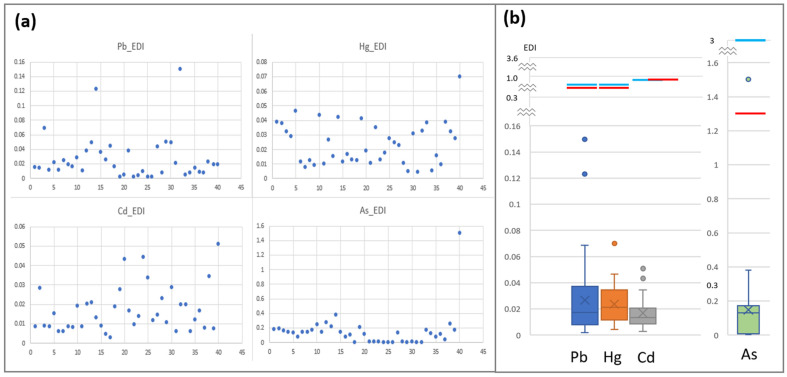
Estimated daily intake (EDI) (µg/kg-bw/day) of lead (Pb), cadmium (Cd), mercury (Hg), and arsenic (As) of infants through breast milk consumption. (**a**) Scatter plots showing the EDI distributions of Pb, Cd, Hg, and As. The *x*-axis represents the infant ID numbers, while the *y*-axis indicates the EDI of each heavy metal in µg/kg-bw/day. (**b**) A box plot showing the EDI of Pb, Cd, Hg, and As in infants consuming breast milk. The *x*-axis corresponds to the different heavy metals, whereas the *y*-axis denotes their respective EDI in µg/kg-bw/day. The blue bars indicate the health-based guidance values from the World Health Organization (WHO) Joint FAO/WHO Expert Committee on Food Additives (JECFA). The red bars indicate the health-based guidance values from the Korea Ministry of Food and Drug Safety (MFDS).

Table 3 displays the concentrations of Pb, Cd, Hg, and As in the breast milk samples of the Korean mothers in this study. Cd was detected in 99% of the samples; its geometric mean (GM) and median value were 0.13 µg/L. The detection rates of Hg, As, and Pb were 97%, 89%, and 79%, respectively. The GM (range) of Hg, As, and Pb was 0.18 µg/L (0.05–1.28), 1.16 µg/L (0.08–9.48), and 0.11 µg/L (0.02–1.49), respectively. Figure 1 shows the distribution of EDI in the breastfed infants. Figure 1a shows the scatterplots illustrating the EDI distributions of Pb, Hg, Cd, and As; meanwhile, Figure 1b depicts the EDI levels of Pb, Cd, Hg, and As. The blue bars indicate health-based threshold values suggested by the JECFA while the red bars indicate the health-based guidance values suggested by the Korean MFDS. The median, GM (SD), and minimum–maximum (min–max) EDI values of Pb were 0.017, 0.016 (0.030), and 0.002–0.150 μg/kg bw day, respectively. The median, GM (SD), and min–max EDI values of Hg were 0.021, 0.019 (0.015), and 0.004–0.070 μg/kg bw day, respectively. The median, GM (SD), and min–max EDI values of Cd were 0.014, 0.014 (0.012), and 0.003–0.051 μg/kg bw day, respectively. The median, GM (SD), and min–max EDI values of As were 0.130, 0.059 (0.238), and 0.003–1.503 μg/kg bw day, respectively (Supplementary Table S2). One EDI value of As exceeded health-based guidance values suggested by the Korea MFDS.

**Figure 2:**
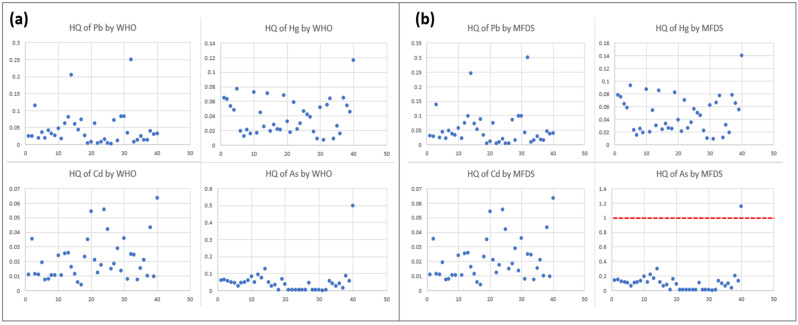
The hazard quotient (HQ) of lead (Pb), cadmium (Cd), mercury (Hg), and arsenic (As) in infants consuming breast milk. (**a**) Scatter plots of the HQ of Pb, Cd, Hg, and As in infants consuming breast milk according to the health-based guidance values from the World Health Organization (WHO) Joint FAO/WHO Expert Committee on Food Additives (JECFA). The *x*-axis represents the infant ID numbers, while the *y*-axis indicates the HQ values for each heavy metal. (**b**) Scatter plots of Pb, Cd, Hg, and As in infants consuming breast milk according to the health-based guidance values from the Korea Ministry of Food and Drug Safety (MFDS). The *x*-axis represents the number of infants, while the *y*-axis indicates the HQ values for each heavy metal. The red dashed line represents a threshold value of 1 for the HQ, indicating a potential health problem.

Figure 2 illustrates the distribution of the HQ values of the infants who were absolutely breastfed throughout the study period according to the threshold values suggested by the JECFA (a) and Korea MFDS (b). As shown in Figure 2, the HQ of As showed the highest value among absolutely breastfeeding infants. In Figure 2b, the red dashed line indicates a threshold value HQ = 1, indicating a potential health risk for the infant. The mean, GM, SD, median, min, and max values of the HQ of Pb, Cd, Hg, and As are presented in Supplementary Table S3.

### 3.3. Association Between Maternal Dietary Intake and HM Levels in Breast Milk

The daily food intake of the lactating mothers according to 16 food groups based on the Korean standard food classification is presented in Supplementary Table S4. Grains had the highest mean value for daily intake (319.29 g/day), followed by vegetables (187.18 g/day), meat (182.41 g/day), fruits (103.4 g/day), and dairy products (86.15 g/day). Table 4 shows the association between the HM concentrations in the breast milk samples and maternal food consumption. The increase in Pb concentration in the breast milk samples was significantly positively associated with the consumption of legumes [β = 0.281, 95% confidence interval (CI) = 0.002–0.029] and seaweed (β = 0.286, 95% CI = 0.001–0.049). Moreover, there were significant positive associations between Cd concentration and the consumption of vegetables (β = 0.218, 95% CI = 0.004–0.036) and seaweed (β = 0.191, 95% CI = 0.001–0.021), Hg concentration and sugar intake (β = 0.351, 95% CI = 0.008–0.059), and As concentration and meat consumption (β = 0.191, 95% CI = 0.002–0.100).

**Figure 3:**
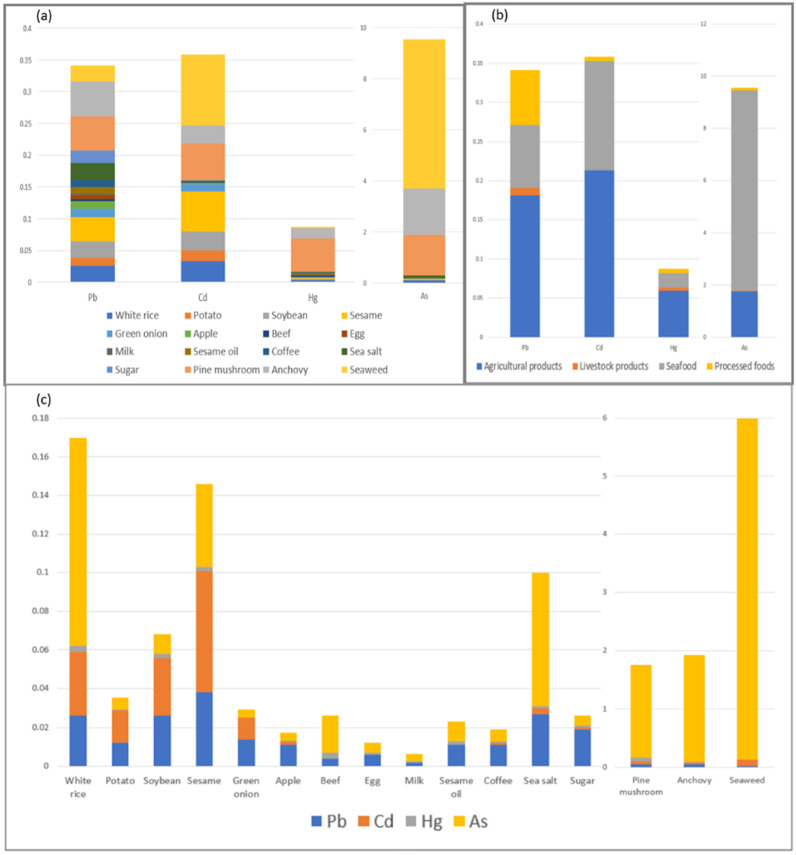
The contribution of maternal food intake to heavy metal exposure and detection rates of heavy metals in foods (**a**) The *y*-axis of the figure represents the concentrations of Pb, Cd, Hg, and As detected in maternal breast milk (µg/L). The color distinctions within the bar graph indicate the relative contribution of maternal intake from different food categories to the concentration of each heavy metal. (**b**) The relative contribution rates of maternal dietary intake, categorized into agricultural products, livestock products, seafood, and processed foods, to the concentrations of Pb, Cd, Hg, and As detected in maternal breast milk. (**c**) The *y*-axis of the figure represents the total concentration of the four heavy metals detected in each food category (µg/L). The color distinctions within the bar graph illustrate the relative proportions of Pb, Cd, Hg, and As.

Figure 3 illustrates the contribution of maternal food intake to the HM concentrations in the breast milk samples and the detection rates of HMs in the food groups. Figure 3a illustrates the contributions of food intake to the concentrations of Pb, Cd, Hg, and As in the breast milk samples. The *y*-axis represents the concentrations of each heavy metal detected in the breast milk samples. Pine mushroom consumption exhibited the most significant contribution to the levels of all HMs, while seaweed consumption contributed the most to Cd and As concentrations. Figure 3b breaks down the contribution rates of agricultural, livestock, seafood, and processed food products. The *y*-axis represents the concentrations of each heavy metal detected in breast milk. The consumption of agricultural products contributed significantly to Pb, Cd, and Hg levels in breast milk, whereas seafood contributed significantly to As levels. Figure 3c shows the relative proportions of Pb, Cd, Hg, and As in representative foods from the 16 food groups. The *y*-axis represents the total concentration of the four HMs detected in each grain group. The color distinctions within the bar graph indicate the relative contribution of each heavy metal. The analysis of HM concentrations in representative high-consumption and high-frequency foods consumed by the lactating mothers revealed relatively high levels of As.

## 4. Discussion

In this study, we assessed the risks posed by HMs in breast milk to infants and found that As was the main contributor to the potential toxicity of breast milk. In addition, frequent consumption of certain food products by the lactating mothers increased the concentration of HMs in breast milk.

The median Pb concentration in the breast milk samples analyzed in this study is significantly lower than values reported in previous studies from Korea (4.11–4.82 μg/L), Iran (45.70 μg/L), and China (1.56 μg/L) [14,23,39]. The median EDI and HQ values of Pb in this study are also lower than those reported in recent studies from China, Iran, and Taiwan [18,23,39]. In a previous study wherein breast milk samples from mothers breastfeeding 2–12 months postpartum were analyzed, the HQ values of Pb exceeded 1 in the breast milk samples of 61 out of 100 participants, indicating potential health risks [23]. In the present study, it was found that the higher the intake of legumes and seaweed by lactating mothers, the higher the Pb concentration in breast milk. Moreover, agricultural products, including soybeans, pine mushrooms, and sesame, were identified as the main contributors of high Pb levels in breast milk. Consistent with the results of this study, the Korean MFDS reported that Pb exposure in agricultural products has increased by 77.6% compared to that in the past [10]. Studies from the United Kingdom and Poland reported that Pb and Cd exceeded the maximum allowable concentrations in agricultural products, particularly in potato skins and dried vegetables [40,41].

The median Cd concentration in the breast milk samples analyzed in this study is similar to that reported in a Chinese study (0.19 μg/L) and lower than the values reported in a Slovakian study (0.36 μg/L) and an Iranian study (0.72 μg/L) [39,42]. The median EDI and HQ values of Cd calculated in this study are higher than those (EDI = 0.005 μg/kg/day, HQ = 0.003–0.014) reported in a recent Chinese study [18] and lower than those reported in studies of Iran, Turkey, China, and Palestine [17,24,39,42]. According to a previous study wherein the health risks of Cd in breast milk to infants under 1 year of age were analyzed by age and gender, the average EDI of Cd in breast milk consumed by infants 1–3 months old was 0.1–0.12 μg/L [42]. Interestingly, from 4 to 12 months of age, the average Cd concentration in the breast milk consumed by both male and female infants significantly increased by 0.61–0.91 μg/L; such an increase was attributed to increased exposure to cosmetics, soil, dust, and food [42]. The EDI value of Cd (0.08 μg/kg/day) reported in another Turkish study [24], wherein 34 breast milk samples were analyzed, is similar to our results. A previous study [24] also revealed that the consumption of vegetables, seaweeds, and grains was positively associated with elevated Cd levels in breast milk. Similar to our results, a previous study [43] reported that the consumption of seaweed, particularly hijiki and nori, can elevate Cd levels, with concentrations reaching 1.196 and 1.005 mg/kg for hijiki and nori, respectively. The consumption of leafy vegetables could be another pathway for Cd exposure [44]. Vegetables are among the most significant sources of Cd exposure, especially in occupationally contaminated regions [44]. Similarly, grains readily absorb and store harmful elements, such as Cd, from the soil as they grow; these harmful metals can then be passed on to humans along the food chain [45]. An increase in Cd levels in breast milk may result from consuming these foods due to the absorption and accumulation of HMs from contaminated environments, such as polluted water and soil [45].

The median Hg concentration level in the breast milk samples analyzed in this study is lower than values reported in previous Korean (0.56–0.61 μg/L), Iranian (2.2 μg/L), and Slovakian studies (0.72 μg/L) [14,46]. The median EDI of Hg calculated in this study is higher than that reported (EDI = 0.001 μg/kg/day) in a recent Chinese study [18]. A previous Iranian study wherein a risk assessment was conducted by separating inorganic Hg, methyl Hg, and total Hg in breast milk samples revealed that 68.5% of the samples exceeded an HQ of 1 for methyl Hg, and 91.1% of the samples exceeded an HQ of 1 for total Hg, indicating potential health risks [46]. Additionally, 96.6% of the samples had HQ values exceeding 1, indicating potential health problems [46]. It is also noteworthy that the HQ for all metals was significantly higher in infants under 6 months than in those 6–12 months of age. The contribution to total HQ followed the order of methyl Hg, inorganic Hg, Cd, and Mn [46]. According to the results of another recent study that analyzed the concentration and health risks of HMs in breast milk consumed by infants 1–12 months of age based on age and sex, values higher than the average in this study were detected across all age groups [42]. The EDI values of Hg in breast milk ranged from 0.26 to 0.47 μg/L for boys and from 0.28 to 0.49 μg/L for girls [42], which are higher than our results. In our study, the consumption of sugar and grains was positively associated with Hg levels in breast milk. Studies that directly link sugar consumption to increased Hg levels are scarce. However, a study from Brazil reported elevated levels of HMs, including Hg, in sugarcane grown in contaminated areas [47]; it also revealed that Hg concentrations were 0.04 mg/kg in sugarcane roots, and Hg levels tend to decrease from roots to stems and leaves. Although we cannot definitively conclude that the sugar consumed by the participants in this study contained high levels of HMs, our results suggest a possible association between sugar consumption and increased HM levels. Grains, such as rice, wheat, and corn, absorb and store Hg and other contaminants from polluted soil, making them potential sources of Hg exposure [45].

The median As concentration reported in this study is higher than values reported in an Iranian study (0.50 μg/L) and a Chinese study (0.86 μg/L) and lower than the value reported in another Iranian study (1.96 μg/L) [23,39,42]. The median EDI value of As calculated in this study is higher than that reported (EDI = 0.051 μg/kg/day, HQ = 0.123–1.694) in a recent Chinese study and lower than that in an Iranian study (EDI range = 0.17–0.32 μg/kg/day) [18,42]. According to the results of an Iranian study, the EDI of As for male and female infants 1–2 months old ranged from 0.3 to 0.32 μg/kg/day, which was higher than that for other age brackets. A previous study [42] also found that HQ values exceeded 1 in 1-month-old male and 2-month-old female newborns, indicating potential health risks. Recently, a previous study [13] revealed that the combined HQ values for As, Al, Cu, Zn, and Fe were significantly high, with an average of 9.40. Among these, the HQ of As was the highest, accounting for 66.59% of the total sum of HQ values [13]. In all of the samples, the EDI value of As exceeded the tolerable daily intake (TDI) [13]. Moreover, food was identified as the likely source of exposure, given the borderline or high levels of As reported in drinking water, bread, and flour throughout the region [13]. In the present study, we found that meat and seafood consumption was associated with increased As concentrations in breast milk. Similarly, a study conducted on Japanese adults demonstrated a significant relationship between fish consumption and elevated As levels [48]. Additionally, a study conducted in Cambodia concluded that fish, rather than drinking water, was the primary source of As exposure in the Cambodian population [49].

Looking at the diets of these mothers in the top 20% of HM HQs in breast milk, the food groups consumed in greater amounts than the overall average were sugars (10.14 g/day), legumes (132.37 g/day), seeds and nuts (14.20 g/day), vegetables (279.26 g/day), mushrooms (12.86 g/day), fruits (137.14 g/day), meats (228.36 g/day), seafood (85.45 g/day), seaweed (12.23 g/day), dairy (90 g/day), oils (10.40 g/day), and condiments (39.05 g/day). Significant differences in food intake compared with the average were observed for legumes, vegetables, fruits, meat, and seafood. As mentioned earlier, all food groups in this study, except fruits, were significantly associated with elevated HM concentrations in the breast milk samples. However, previous studies have suggested that fruits such as pineapples, mangoes, and cherries can be considered sources of HMs, including Pb, Cd, Hg, and As [50,51]. Thus, food can be a source of HM exposure through various pathways, and its impact can be influenced by various factors such as soil environment, regulatory policies, industrial processes, and cultural consumption habits, which vary by region or country. Therefore, caution should be exercised when collecting and interpreting data.

Among the HMs analyzed in this study, As contributed the most to the HQ, followed by Hg, Cd, and Pb. These results are consistent with those of a recent Iranian study, where As and Hg contributed significantly to elevated HQ levels [13]. Similarly, a previous study in Saudi Arabia that assessed the risks of Pb, Hg, Cd, and Mn reported that the combined HQ of the four metals exceeded 1, indicating potential health risks, with Hg being the largest contributor [46]. The reason for the high HQ values of As and Hg is understood to be the similarities in the diets of Korea, Saudi Arabia, and Iran. Korea, Iran, and Saudi Arabia have similar diets primarily consisting of grains, such as rice and wheat, and meat, such as chicken, beef, and fish [52]. These dietary characteristics can significantly affect exposure to As and Hg [52]. Rice has a strong tendency to absorb As and Hg because it is cultivated in flooded environments [53]. This waterlogged condition allows As to dissolve from the soil and be easily absorbed by plant roots [53,54]. In particular, it can move easily in a low-oxygen environment, such as waterlogged soil, and is transferred to the grain portion of rice [55]. The rice root structure and metabolic processes make it highly effective at absorbing As compounds. Similarly, wheat can absorb As and Hg when exposed to contaminated soil [52]. Past pesticide use, industrial pollution, and water and soil contamination play crucial roles in the absorption of pollutants by grains [53,54,55].

This study has several limitations. First, this is a cross-sectional study; only one breast milk sample was collected from each mother, making it difficult to reflect internal or external variability within individuals. Second, Hg and As were not separated into more toxic forms, such as methyl-Hg and inorganic As, when their concentrations were calculated. Future studies should incorporate chemical speciation along with larger sample sizes to improve statistical power and provide a more precise evaluation of exposure levels and health risks associated with these toxicants. Nevertheless, the strengths of this study were the analysis of a considerably large nationwide sample compared with previous studies and the implementation of a realistic risk assessment by individually assessing each infant’s individual body weight and daily breast milk intake.

## 5. Conclusions

In this study, we investigated the relationship between the concentrations of HMs in the breast milk of Korean mothers and their dietary habits, as well as the potential health risks to infants consuming breast milk and the relative contribution of each metal. It is crucial for mothers to reduce HM exposure through dietary adjustments that can help decrease the concentration of these HMs in breast milk. By paying closer attention to their daily habits, mothers can minimize the potential risks of breastfeeding, such as HM contamination, while maximizing the well-known benefits of superior nutrition and immunity compared with formula. This, in turn, contributes to the health and well-being of future generations, provides essential insights for both the general public and breastfeeding mothers, and aids in the development of food safety regulations, the establishment of policy guidelines, and the design of educational interventions. This comprehensive approach will improve public health outcomes and promote safer breastfeeding practices.

## Figures and Tables

**Table 1:** Health-based guidance values suggested by Joint FAO/WHO Expert Committee on Food Additives (JECFA) and Korea Ministry of Food and Drug Safety (K-MFDS).

Heavy Metals	JECFA	K-MFDS
Pb	0.6 µg/kg/day (loss of 1 IQ point in children) ^a^	BMDL_0.1_ 0.5 µg/kg bw/day ^e^ (developmental toxicity in children)
Cd	PTMI 25 µg/kg bw/month ^b^ (0.8 µg/kgbw/day)	PTMI 25 µg/kg bw/month ^f^
Hg	Inorganic Hg: PTWI 4.0 µg/kg bw/week (Kidney weight change) ^c^ (0.6 µg/kgbw/day)	Inorganic Hg: TWI 3.7 µg/kg bw/week ^g^ (0.5 µg/kgbw/day)
As	BMDL_0.5_ 3.0 µg/kg bw/day (lung cancer) ^d^	Inorganic As: PTWI 9.0 µg/kg bw/week ^h^ (1.3 µg/kgbw/day)

Pb, lead; Cd, cadmium; Hg, mercury; As, arsenic; PTMI, provisional tolerable monthly intake; PTWI provisional tolerable weekly intake; ^a^ JECFA (2011); ^b^ JECFA (2021); ^c^ JECFA (2011); ^d^ JECFA (2011); ^e^ K-MFDS (2011); ^f^ K-MFDS (2023); ^g^ K-MFDS (2013); ^h^ K-MFDS (2014).

**Table 2:** Socioeconomic characteristics of participants (*n* = 209).

Variables	Categories	*n*/M	%/SD	Median	Min	Max
Maternal age (years)		34.82	4.02	35	22	43
Maternal BMI (kg/m^2^)		23.23	3.60	22.60	15.99	39.21
Education	<college	11	5.26			
	≥college	198	94.74			
Household income (USD/month)						
	≤5000	89	42.58			
	>5000	120	57.42			
Employment status	Yes	105	50.24			
	No	104	49.76			
Residence area	Seoul-Gyeonggi	111	53.11			
	Gangwon	44	21.05			
	Chungcheong	15	7.18			
	Honam-Jeju	11	5.26			
	Yeongnam	28	13.40			
Parity	Primipara	126	60.29			
	Multipara	83	39.71			
Infant age (months)		2.47	1.55	2.27	0.10	5.67
Infant sex	Boys	101	48.33			
	Girls	108	51.67			
Weight (kg)		5.71	1.56	5.60	2.99	9.40
Height (cm)		58.81	6.35	58.00	49.00	84.00
Weeks at birth		38.71	1.27	38.6	34.4	41.2
Birth height (cm)		50.46	2.64	51	36.5	57.3
Birth weight (kg)		3.19	0.38	3.17	2.18	4.38
Absolute breast milk feeding (*n* = 40, mL/day)		883.54	226.01	900	420	1260

M, mean; SD, standard deviation; Min, minimum; Max, maximum; BMI, body mass index.

**Table 3:** Concentrations of heavy metals in breast milk (*n* = 209).

Analytes	LOD (µg/L)	DF (%)	GM	Min	P5	P25	P50	P75	P95	Max
Pb	0.017	165 (79)	0.11	0.02	0.03	0.07	0.10	0.17	0.57	1.49
Cd	0.030	207 (99)	0.13	0.04	0.05	0.08	0.13	0.20	0.45	1.51
Hg	0.047	204 (97)	0.18	0.05	0.06	0.11	0.19	0.27	0.56	1.28
As	0.054	187 (89)	1.16	0.08	0.32	0.76	1.11	1.72	4.62	9.48

Pb, lead; Cd, cadmium; Hg, mercury; As, arsenic; LOD, limit of detection; DF, detection frequency; GM, geometric mean; Min, minimum; Max, maximum; P, percentile.

**Table 4:** Association between concentration of heavy metals in breast milk and maternal dietary intake.

Variables	Pb		Cd		Hg		As	
	β	95%CI	β	95%CI	β	95%CI	β	95%CI
Grains	−0.018	−0.065–0.055	−0.029	−0.041–0.031	−0.126	−0.089–0.027	0.026	−0.175–0.220
Potato starches	0.143	−0.005–0.022	−0.089	−0.012–0.005	−0.169	−0.022–0.004	−0.135	−0.070–0.018
Sugars	0.014	−0.025–0.028	−0.152	−0.027–0.006	**0.351**	**0.008–0.059**	0.108	−0.052–0.125
Legumes	**0.281**	**0.002–0.029**	−0.147	−0.013–0.003	−0.015	−0.014–0.012	−0.023	−0.048–0.039
Nuts and seeds	−0.054	−0.030–0.019	−0.147	−0.025–0.004	−0.189	−0.042–0.005	0.023	−0.072–0.088
Vegetables	0.107	−0.018–0.148	**0.218**	**0.004–0.036**	0.058	−0.025–0.040	0.015	−0.103–0.117
Mushrooms	0.092	−0.012–0.030	0.043	−0.010–0.016	−0.070	−0.027–0.014	−0.054	−0.086–0.051
Fruits	0.079	−0.007–0.014	0.014	−0.006–0.007	−0.085	−0.014–0.006	0.121	−0.015–0.052
Meat	−0.192	−0.036–0.004	0.072	−0.008–0.016	0.028	−0.017–0.022	**0.191**	**0.002–0.100**
Eggs	−0.145	−0.024–0.006	−0.012	−0.009–0.008	−0.148	−0.023–0.006	0.006	−0.047–0.050
Fish and shellfish	0.041	−0.012–0.017	0.072	−0.006–0.012	−0.070	−0.018–0.010	0.074	−0.034–0.063
Seaweed	**0.286**	**0.001–0.049**	**0.191**	**0.001–0.021**	0.018	−0.022–0.025	−0.005	−0.080–0.077
Dairy products	0.070	−0.007–0.014	−0.031	−0.007–0.005	0.007	−0.010–0.010	−0.067	−0.044–0.024
Oils and fats	−0.152	−0.075–0.026	0.142	−0.016–0.045	−0.111	−0.065–0.032	0.047	−0.142–0.190
Beverages	−0.046	−0.014–0.009	−0.076	−0.010–0.005	−0.150	−0.019–0.004	0.073	−0.027–0.052
Seasonings	−0.157	−0.063–0.016	−0.023	−0.026–0.022	0.017	−0.036–0.040	0.076	−0.093–0.165

## Data Availability

The data presented in this study are available on request from the corresponding author due to participant confidentiality.

## Supplementary Materials

The following supporting information can be downloaded at: https://www.mdpi.com/article/10.3390/toxics13050381/s1, Table S1. Detailed operating conditions in the analysis of breast milk samples. Table S2. Estimated daily intake (EDI) of heavy metals in Korean breast-feeding infants (µg/kg bw/day). Table S3. Risk assessment of breast milk feeding infants according to the health-based guidance values by WHO JECFA and Korea MFDS. Table S4. Amount of food intake of breast milk feeding mothers in Korea (g/day) (*n* = 103).

## Abbreviations

The following abbreviations are used in this manuscript:

HM	heavy metal
MFDS	Ministry of Food and Drug Safety
tAs	total arsenic
ICP-MS	inductively coupled plasma mass spectrometry
DRC	dynamic reaction cell
DMA	direct mercury analyzer
LOD	limit of detection
TDI	tolerable daily intake
EDI	estimated daily intake
HQ	hazard quotient
GM	geometric mean
