# Supplementary material for: The Association Between Maternal Dietary Intake and the Risk of Heavy Metals in Human Breast Milk in Korea
Source: Toxics. 2025 May 8;13(5):381. doi: 10.3390/toxics13050381 (PMC12115469; doi:10.3390/toxics13050381)
Supplement: Supplementary file 1 [file toxics-13-00381-s001.zip › toxics-3588785-supplementary.pdf]

Supplementary Table S1. Detailed operating conditions in the analysis of breast milk samples

ITEM	Operating condition
ICP-MS Model	NeXION 2000B
RF power	1500 ~ 1600 W
Nebulizer gas flow	0.8 ~ 1.05 (optimized daily)
Plasma gas flow	14 ~ 16 L/min
Auxiliary gas flow	1.1 ~ 1.3 L/min
Measurement units	ng/mL
Spray chamber	Cyclonic
Interface	Pt cone
Scan mode	Peak hoping

Supplementary Table S2. Estimated daily intake (EDI) of heavy metals in Korean breast-feeding infants (μg/kg bw/day)

	Mean	GM	SD	Median	Min	Max
Pb	0.027	0.016	0.030	0.017	0.002	0.150
Cd	0.017	0.014	0.012	0.014	0.003	0.051
Hg	0.024	0.019	0.015	0.021	0.004	0.070
As	0.148	0.059	0.238	0.130	0.003	1.503

GM, geometric mean; SD, standard deviation; Min, minimum; Max, maximum;

Supplementary Table S3. Risk assessment of breast milk feeding infants according to the health based guidance values by WHO JECFA and Korea MFDS

	JECFA						Korea MFDS					
	Mean	GM	SD	Median	Min	Max	Mean	GM	SD	Median	Min	Max
HQ of Pb	0.044	0.027	0.051	0.029	0.003	0.250	0.053	0.032	0.061	0.035	0.004	0.300
HQ of Cd	0.021	0.017	0.015	0.017	0.004	0.064	0.021	0.017	0.015	0.017	0.004	0.064
HQ of Hg	0.040	0.032	0.024	0.035	0.007	0.117	0.047	0.039	0.029	0.042	0.009	0.140
HQ of As	0.049	0.020	0.079	0.043	0.001	0.501	0.114	0.045	0.183	0.100	0.002	1.156

HQ, hazard quotient; Pb, lead; Cd, cadmium; Hg, mercury; As, arsenic; JECFA, Joint FAO/WHO Expert Committee on Food Additives; MFDS, Ministry of Food and Drug Safety; GM, geometric mean; SD, standard deviation; Min, minimum; Max, maximum;

Supplementary Table S4. Amount of food intake of breast milk feeding mothers in Korea (g/day) (n=103)

Variables	Mean	SD	Median	Min	Max
Grains	319.29	151.49	290	79.20	1045
Potato starches	33.71	68.40	0	0	350
Sugars	7.31	8.78	3.50	0	39.5
Legumes	58.86	90.31	10	0	400
Nuts and seeds	4.63	12.19	0	0	60
Vegetables	187.18	106.37	176.17	2.32	495.4
Mushrooms	5.18	18.18	0	0	150
Fruits	103.40	145.47	10	0	663
Meat	182.41	139.27	140	0	740
Eggs	27.85	36.86	8	0	144
Fish and Shellfish	58.05	82.36	22.5	0	518
Seaweed	10.29	17.24	3	0	103
Dairy products	86.15	125.58	0	0	570
Oils and Fats	10.01	6.95	9	0	40
Beverages	69.95	169.07	0	0	1000
Seasonings	30.47	39.81	24.4	1.52	372.2

SD, standard deviation; Min, minimum; Max, maximum, BMI, body mass index; Pb, lead; Cd, cadmium; Hg, mercury; As, arsenic; β, standardized coefficient; CI, confidence interval, Multiple linear regression after adjusting covariates including maternal body mass index, education, job, parity, income, region.
